# Mechanisms of tactile sensory deterioration amongst the elderly

**DOI:** 10.1038/s41598-018-23688-6

**Published:** 2018-04-19

**Authors:** Lisa Skedung, Charles El Rawadi, Martin Arvidsson, Céline Farcet, Gustavo S. Luengo, Lionel Breton, Mark W. Rutland

**Affiliations:** 1 RISE Research Institutes of Sweden, Bioscience and Materials, Stockholm, SE-114 28 Sweden; 2 L’Oréal Research and Innovation, Aulnay-sous-Bois, 93600 France; 3 KTH Royal Institute of Technology, Surface and Corrosion Science, Stockholm, SE-100 44 Sweden

## Abstract

It is known that roughness-smoothness, hardness-softness, stickiness-slipperiness and warm-cold are predominant perceptual dimensions in macro-, micro- and nano- texture perception. However, it is not clear to what extent active tactile texture discrimination remains intact with age. The general decrease in tactile ability induces physical and emotional dysfunction in elderly, and has increasing significance for an aging population. We report a method to quantify tactile acuity based on blinded active exploration of systematically varying micro-textured surfaces and a same-different paradigm. It reveals that elderly participants show significantly reduced fine texture discrimination ability. The elderly group also displays statistically lower finger friction coefficient, moisture and elasticity, suggesting a link. However, a subpopulation of the elderly retains discrimination ability irrespective of cutaneous condition and this can be related to a higher density of somatosensory receptors on the finger pads. Skin tribology is thus not the primary reason for decline of tactile discrimination with age. The remediation of cutaneous properties through rehydration, however leads to a significantly improved tactile acuity. This indicates unambiguously that neurological tactile loss can be temporarily compensated by restoring the cutaneous contact mechanics. Such mechanical restoration of tactile ability has the potential to increase the quality of life in elderly.

## Introduction

In common only with taste, touch is based on the intimate contact of a body surface with a material and as with all senses, tactile perceptual ability declines with age^1–4^. Human hands and fingers are used for active exploration of our surroundings as well as for grasping objects^5,6^ and are our primary “tactile probes”; fingertip interactions are thus highly relevant for understanding tactile perception.

The so-called “sensorial” evaluation of touch (the association of sensations with subjective descriptions) is a well-established field^7^ and is paramount when communicating about touch; for example in consumer panel studies^8,9^. Although the importance of physical quantities such as roughness, elastic modulus *etc*. is commonly accepted, a clear-cut understanding of their individual impact on perception is lacking since it is difficult to vary one parameter independently^10–13^. There is an increased interest recently on methods based on stimuli detection; such psycho-physical techniques provide a complementary approach and rely on objective tests, such as whether a difference can be detected^14^. In general, such approaches provide quantitative data that can be compared to, and ideally correlated with, individual physical quantities or combinations thereof. Skedung *et al*.^15^ observed, for example, how the friction coefficient diminished with increased average roughness on printing papers and explored the perceived similarities of calibrated wrinkled surfaces of different wavelengths and amplitudes^10^. Friction coefficient and pattern wavelength were directly linked to the generated tactile space and it was also found that amplitudes as small as 15 nm could be distinguished from blank surfaces. Furthermore, although the finger friction coefficient is the *physical* parameter which varies, the applied load is unconsciously regulated to maintain an optimal friction force^10,15^. Thus it is likely that from a *perceptual* perspective it is the applied load which is monitored and registered. It is known that roughness-smoothness, hardness-softness and stickiness-slipperiness are predominant *perceptual* (as opposed to physical) dimensions in macro texture perception^14,16–18^. This perceptual dimensionality remains intact when the texture scale is reduced to the micro- and nanoscale^19^. It is not clear whether the tactile discrimination ability for these textures remains intact with age.

Skin can be considered a biocomposite material of multiple layers. Consideration of its structure is essential for understanding the generation and transmission of vibrations across the tissue, which are detected by the cutaneous receptors underpinning our somatosensory system^5,20^ and may well be amplified by fingerprints^21^. The influence of the dermis, epidermis and subcutaneous tissue has been widely studied in relation to the induced deformation and the friction observed^22–24^. While less studied, there is increasing evidence of the importance of the harder stratum corneum layer for friction^25^. Friction forces depend on the real area of contact, and thus the local deformability of the stratum corneum properties impacts this parameter^19,26^. Lévêque *et al*.^3^ for example, investigated how the hydration of this layer influences its elasticity and demonstrated improved, static, cheek spatial acuity using the two-point discrimination gap method. Other attributes of the skin surface are also known to play a role in skin friction^27^, such as the micro relief^12^, or the presence of sweat and sebum^25^.

Life expectancy is increasing^28^, yet few studies have explored the limits of tactile perception in the elderly. Age is known to affect both the mechanical and physical properties of the skin as well as neurophysiological capabilities for the detection, transmission or interpretation of touch signals^1,3,29–32^. The situation is complicated by the fact that the 2-point threshold approach used in many studies addresses *static* touch, but there is evidence that *dynamic* touch is affected differently^33^. The relative extent to which the two potential contributions (mechanical or neural) impact sensory perception is unknown. In the correction of sensory decline in the senses of sight and hearing, however, which also deteriorate with age in a well-documented fashion^34,35^, the *mechanical*, rather than the neurological deficiency, is systematically addressed: Spectacles correct for the deformation of the cornea and hearing aids amplify audio signals. Thus the questions can be posed as to whether i) there is a similar reduction in tactile sensory discrimination of fine texture with increasing age, ii) this deterioration can be attributed to age related changes in skin mechanical properties and iii) such mechanical deficiencies can be analogously remediated.

An earlier technical protocol using *wrinkled* surfaces^10,19^ has thus been adapted to study in detail the changes in tactile perception ability that occur at advanced age using active touch on fine textures. The abilities of young and elderly participants to haptically discriminate between different, well defined patterned textures have been quantified, and compared to physical properties of the skin (finger friction, finger hydration and elasticity).

## Results and Discussion

### Lower tactile discrimination ability in the elderly group

Six systematic textures varying in surface pattern wavelength have been fabricated by an established method^10^. They were used in a tactile perception test where the task was to judge whether a presented surface was perceived as the same or different to a reference surface. The nominal wavelengths of the test surfaces used in this same-different tactile perception test were 20 µm, 40 µm, 60 µm, 80 µm (denoted S20, S40, S60 and S80, respectively), 100 µm (Ref100) as well as a blank, smooth surface with no systematic texture (S0). Ref 100 is closest to the limit of what can be considered “fine texture”^19^. A pilot study with 10 elderly and 10 young female subjects indicated that the young group hit a threshold at 60 µm *i.e*., that the difficulty of the task increased significantly for this wavelength and that S60 could not reliably be distinguished from Ref100. The elderly group had a much lower rate of successful task completion even at 20 µm. The results below are obtained for a larger study consisting of 30 “young” (19–25 y) and 30 elderly female subjects (67–85 y), none of whom participated in the pilot. Six repeated tactile perception comparisons to the reference were performed for each test surface and participant, and were presented and evaluated in a unique randomized order (in total 36 comparisons in each perception test).

**Figure 1:**
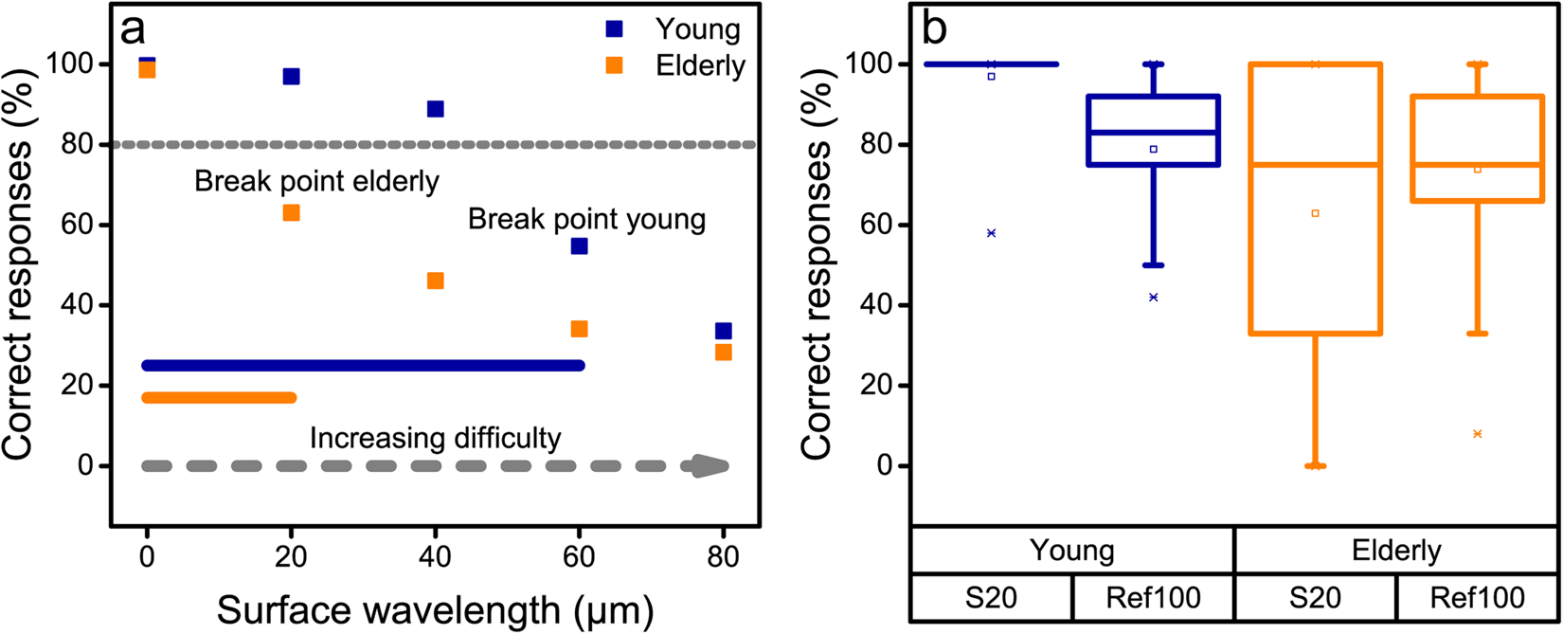
Tactile discrimination ability for the two groups. (**a**) Proportion of correct answers in identifying whether the stimulus was different to the reference sample Ref100 (100 µm in wrinkle wavelength). The difficulty of the task clearly increases for both groups as the wavelength of the stimulus texture approaches that of the reference. The “break point” (defined as when the success rate falls below 80%) is seen for S20 (20 µm) for the elderly group and for S60 (60 µm) for the young group (N = 30 for both groups). (**b**) Box plot of the means from each participant in the two groups (N = 2 × 30), showing the tactile discrimination ability of S20 versus Ref100 as well as the correct responses for Ref100 versus itself (meaning “same” as the correct answer). Since the young and the elderly groups have a very similar proportion of correct responses for the latter task, the tendency to guess “different” is the same for both groups when the task is perceived as difficult.

The averaged results are displayed in Fig. 1 as percentage of correct responses as box plots, showing the interquartile range IQR (25^th^ and 75^th^ quartile = 50% of the data) with the mean (square), median (line), whiskers indicating variability within 1.5 IQR, as well as outliers. An answer was held to be correct when the test surface was judged different from the reference or when the reference was judged the same when presented against itself. The smooth surface has “perfect” scores of 100% and 99% for the young and elderly, respectively, indicating that it was easily detected as different from Ref100. This can be attributed to the greater real contact area between surface and finger during tactile interaction compared with all the other surfaces and a correspondingly increased friction coefficient^22^. The young group successfully differentiated S20 and S40 from Ref100 (average correct response of 97% and 89%, respectively), and the drop in tactile discrimination ability was seen with S60 (55%). S80 is even closer to the reference wavelength of 100 µm and not unexpectedly was even harder to distinguish (34%). The drop in discrimination ability for the elderly group is already observed for S20 where the number of correct responses is 63% and therefore below the 80% criterion for high performance (see below).

For the comparison of Ref100 with itself, *i.e*. where “same” is the correct answer, the young and elderly groups showed a similar correct response level of 79% and 76%, respectively. The fact that this score is not 100% indicates a tendency to answer “different” when the participant is uncertain. This tendency was thus factored into the decision to set the successful detection criterion to 80% correct responses. It is the same for both groups and should not represent a bias in the group comparison.

The tactile discrimination ability (S20) was significantly lower for the elderly group compared to the young. For the young group, the mean value was 97%, with a standard deviation (s.d.) of 17%. For the elderly group, the corresponding values were mean = 63%, s.d. = 48% (F(1,58) = 26.65, p < 0.001). Only two individuals in the young group failed at reliably distinguishing S20 from Ref100, whereas 17 among the elderly failed the same task. For S60 and S80 both groups showed difficulties in perceiving differences to the reference (<80% correct responses).

It is thus abundantly clear that dynamic touch on fine textures exhibits an age-related decline in discrimination ability which may well be related to the decrease in tactile discrimination sensitivity with age reported for static touch^1–4^ and object discrimination^3,36^. The physical dimensions underpinning the *active* touch discrimination of fine textures have previously been identified^10^ as being associated with the friction coefficient (and the resulting finger loading^15^), and the wavelength of the surfaces. It seems likely that the former depends on responses from slow adapting receptors (deformation) whereas the latter depends on vibrations detected by the fast adapting Pacinian receptors^37,38^. It would thus seem logical to start with frictional, or “biotribological”, studies to identify the cause of the active touch deterioration. Skin biomechanics affect these properties strongly, and there may be a direct link to the trends seen for static touch, which is also highly dependent on biomechanical properties. This has been done under an ensuing heading.

**Figure 2:**
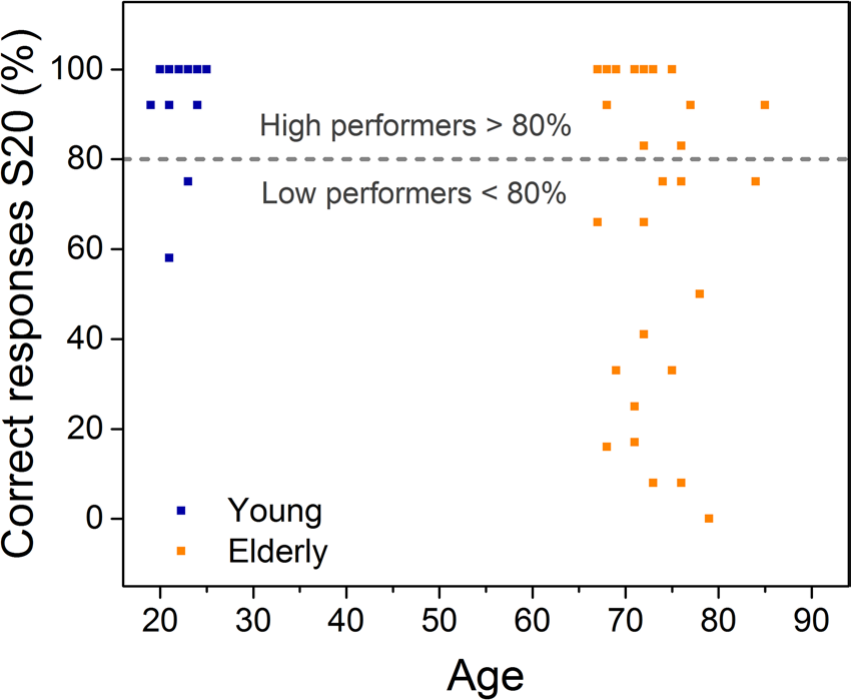
Not all elderly subjects show a decrease in tactile discrimination ability. Average correct responses for S20 of all 60 subjects, showing that 13/30 from the elderly group perform at the same level as the young. The criterion of 80% correct responses is used to separate high and low performers. Age is plotted on the abscissa as a convenient means to separate the two groups.

Before leaving the same-difference test however, it remains to identify an additional important finding. Individual examination of the correct response distribution of all 60 participants (for S20) in Fig. 2, clearly reveals that a significant fraction of the elderly group displays unimpaired ability. This bimodal distribution needs to be considered in future studies of age-related perception decline. The retention of an active touch tactile acuity has earlier been reported in aged, blind individuals^39^, possibly indicating that retention is associated with use, though this speculation is beyond the scope of this work. Using the criterion of 80% success as the demarcation between successful and unsuccessful tactile discrimination ability, the elderly participants could be divided into a high performing sub-group (13 individuals) and a low performing sub-group (17). Thus approximately 43% of elderly group performed at the same level as the young. A direct implication of this observation is that future studies aimed at identifying means to improve age related texture discrimination should address this distribution and adopt an appropriate recruitment strategy.

### Bio-mechanical and bio-tribological differences in young and aged skin

**Figure 3:**
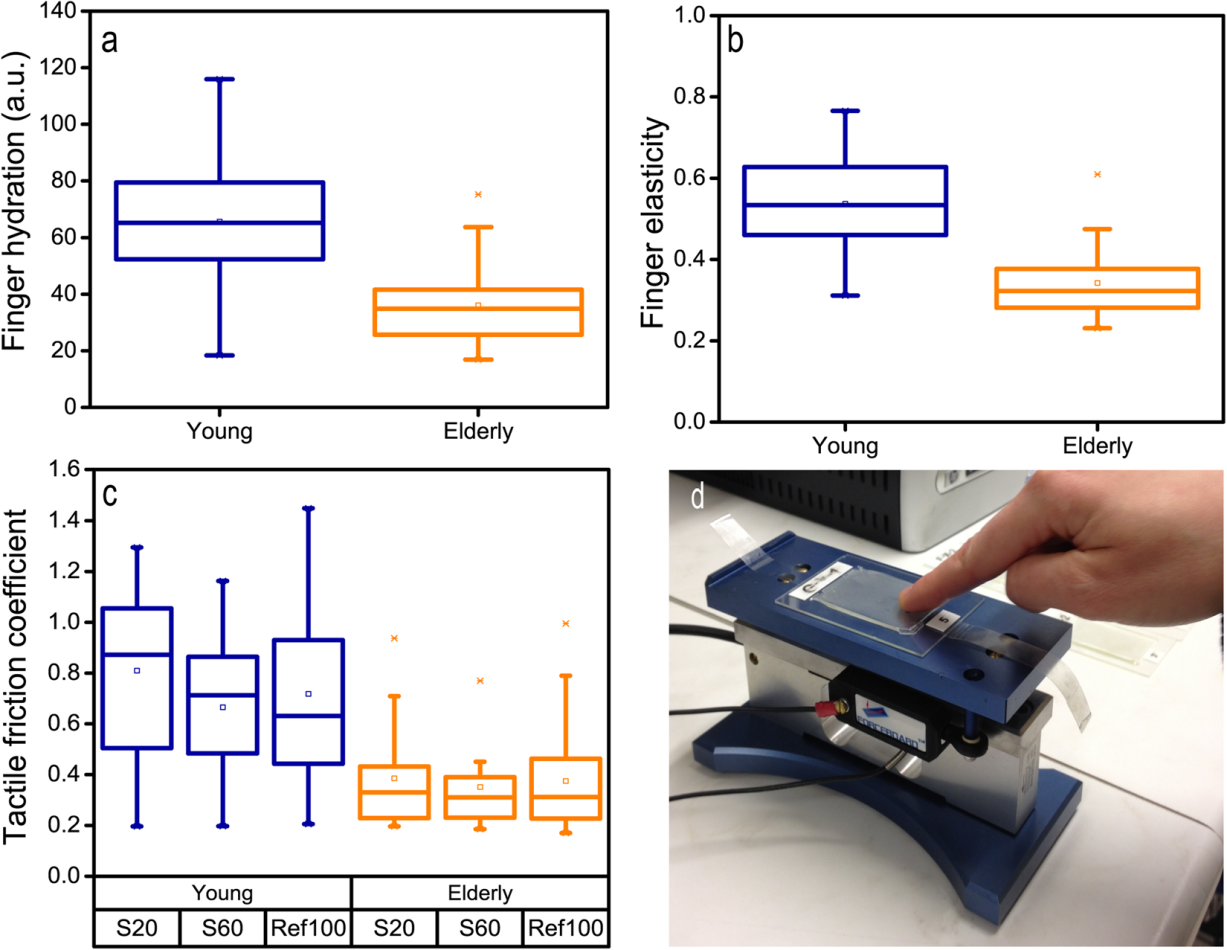
The groups display significantly different bio-mechanical and bio-tribological values. The elderly group display (**a**) lower finger hydration (**b**) lower finger elasticity and (**c**) lower tactile friction coefficients obtained by the continuous recording of friction force and applied load upon interaction using a (**d**) ForceBoard.

Figure 3 illustrates differences in finger elasticity, finger hydration and tactile friction between the young and elderly groups. A one-way ANOVA indicates that the finger hydration level is significantly lower among the elderly group compared to the young (young: mean = 66 a.u., s.d. = 23 a.u.; elderly: mean = 36 a.u., s.d. = 14 a.u.) (F(1,59) = 36.14, *p* < 0.001). Skin elasticity is determined by extracting various parameters from measurements of skin deformation as a function of time. The supporting information provides details of the technique (see also Fig. S1 and Table S1) whereas Fig. S2 shows how the parameters vary between the two groups. The elastic recovery parameter is here used as the measure of finger elasticity since it has earlier been identified as the relevant parameter for describing age related changes^40–42^. Note, that it is not an estimate of elastic modulus, but rather the reversibility of applied deformations; this (unitless) finger elasticity is significantly lower in elderly skin (young: mean = 0.54, s.d. = 0.12; elderly: mean = 0.34, s.d. = 0.08) (F(1,54) = 49.16, p < 0.001). Aged skin is normally reported as drier and less elastic than young skin^42–44^.

Friction between a finger and the textured surfaces, (“tactile friction”)^10,15,19^ was measured in a continuous reciprocating manner on surfaces S20, S60 and Ref100 (see Fig. 3d). The resulting friction coefficients (here, the ratio between friction force and applied load) are shown in Fig. 3c. A two-way repeated ANOVA was used to assess the significance of differences between the average tactile friction coefficients on the three surfaces and the two age groups (N = 2*29). A Tukey *post hoc* test shows that the young group possesses a significantly higher tactile friction coefficient on all three surfaces (*p* < 0.001). The large standard deviations reflect the well-known individual spread in tactile friction between individuals^15,19,23^, and consequently, no significant differences are observed between the three surfaces at group level. However *individual differences* indicate that the young volunteers show a slightly greater difference in tactile friction coefficient between surfaces S20 and S60 compared to the elderly volunteers. In both groups, the majority show highest tactile friction coefficient on the smallest wavelength surface (59% of the elderly and 69% of the young). The higher friction level of the young volunteers is almost certainly an effect of the higher finger hydration level. Such a relation between skin hydration and skin friction is well reported in the literature^19,23,45^.

As stated in the introduction, the friction coefficient is unconsciously used to moderate the applied load (how hard the finger is pressed) and this may well be the perceptual prompt. The elderly do indeed press somewhat harder as a result of the lower friction coefficients experienced though the variation at the individual level is large) and the data is displayed in the SI. (Note that the load is not recorded during perceptual experiments, only during tactile friction measurements).

**Figure 4:**
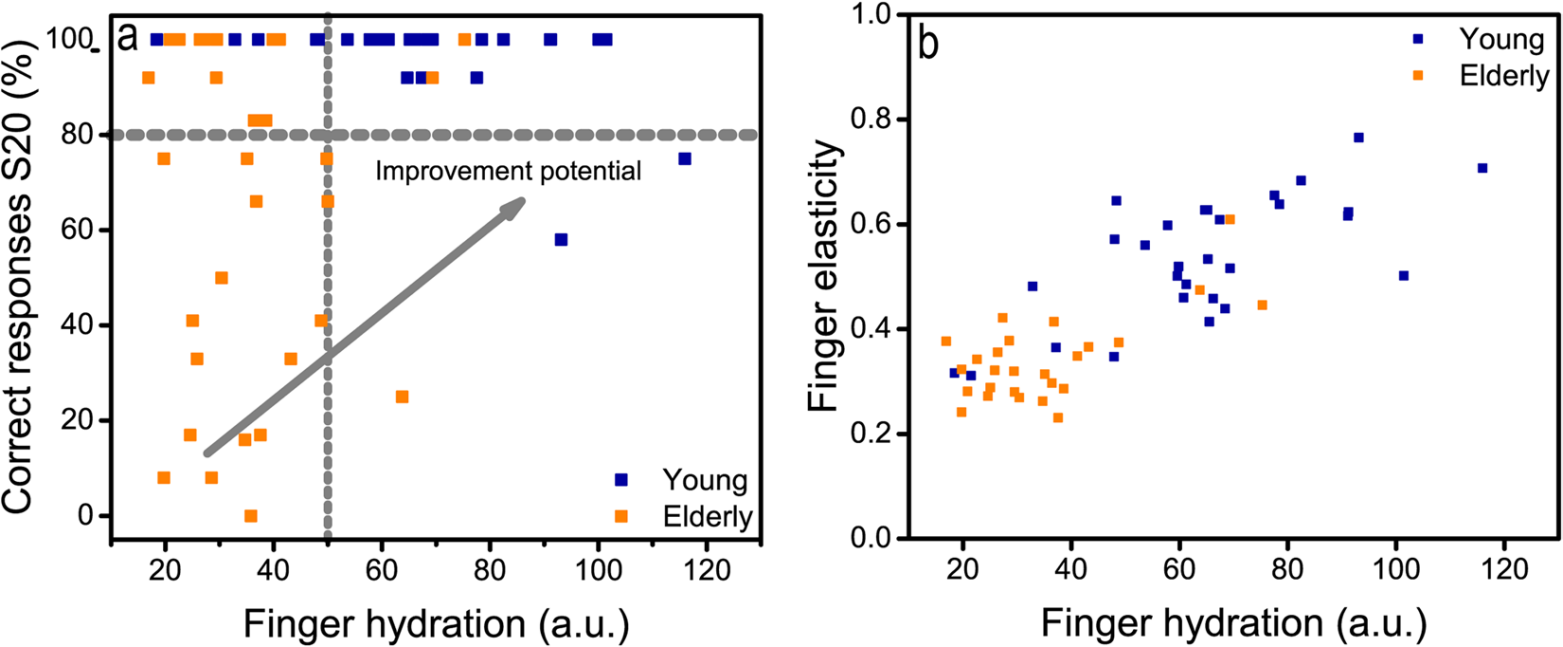
Biomechanical properties and tactile ability. (**a**) Scatter plot showing the correct responses on S20 (perceiving S20 as different from Ref100). There is improvement potential for the subjects below 80% correct responses. (**b**) Finger elasticity appears to increase with increasing hydration.

In order to evaluate a possible link between hydration and tactile perception ability, individual correct responses from all volunteers for S20 are plotted in Fig. 4 versus the individual finger hydration value. As can be seen, all the young participants return above 80% correct responses and the elderly participants are scattered over the entire possible interval from 0% to 100% correct responses. An interesting observation is that above a hydration level of 50 a.u, an almost exclusively high performance is observed, whereas at lower levels of hydration, both high (>80%) and low (<80%) performers are found. A similar plot is obtained with finger elasticity, (not shown) but as can be seen in Fig. 4b, finger elasticity is clearly related to the finger hydration level. These parameters would thus appear to be degenerate and indistinguishable in terms of their intrinsic relevance. From a contact mechanics perspective, it is the elastic modulus that affects the true area of contact and thus the friction coefficient. Skin is a multilayer structure and the macroscopic deformation of the finger corresponds to an elastic modulus on the scale of kPa. This is effectively maximised at very low loads, below 1 N. The friction coefficient, and the area of contact are dependent on local deformations of the Stratum Corneum (SC)^26^ which has a much higher elastic modulus (ca 0.005–0.1 GPa)^46^As mentioned before, the elasticity parameter is not a direct measure of the elastic modulus, but the elastic modulus of the SC also has a strong, inverse, dependence on the moisture content (as well as the scale of the deformation)^46,47^, so the parameters are unambiguously linked. At very high moisture contents the SC also exhibits plastic deformations which lead to even higher contact areas and friction coefficients, and also lower elastic reversibility – though this depends strongly on how the parameters are defined (see SI). In addition, at higher humidity/moisture content there is the possibility for capillary condensation^48^ and/or occlusion^45^ of liquid water in the contact. This also contributes to increased friction. The role of moisture on friction is further discussed in the next section.

Clearly, a hypothesis to test is whether an increase or improvement in finger hydration could improve the dynamic tactile discrimination ability in the elderly group, analogously to observations for static touch, where the application of a moisturizer containing 5% of glycerol increased both the hydration and static touch acuity^3^. Thus, to evaluate a possible improvement in tactile discrimination and changes in cutaneous properties, the group of 30 elderly subjects were divided into a high performing (N = 13) and a low performing (N = 17) sub-group.

### Improvement in tactile ability

**Figure 5:**
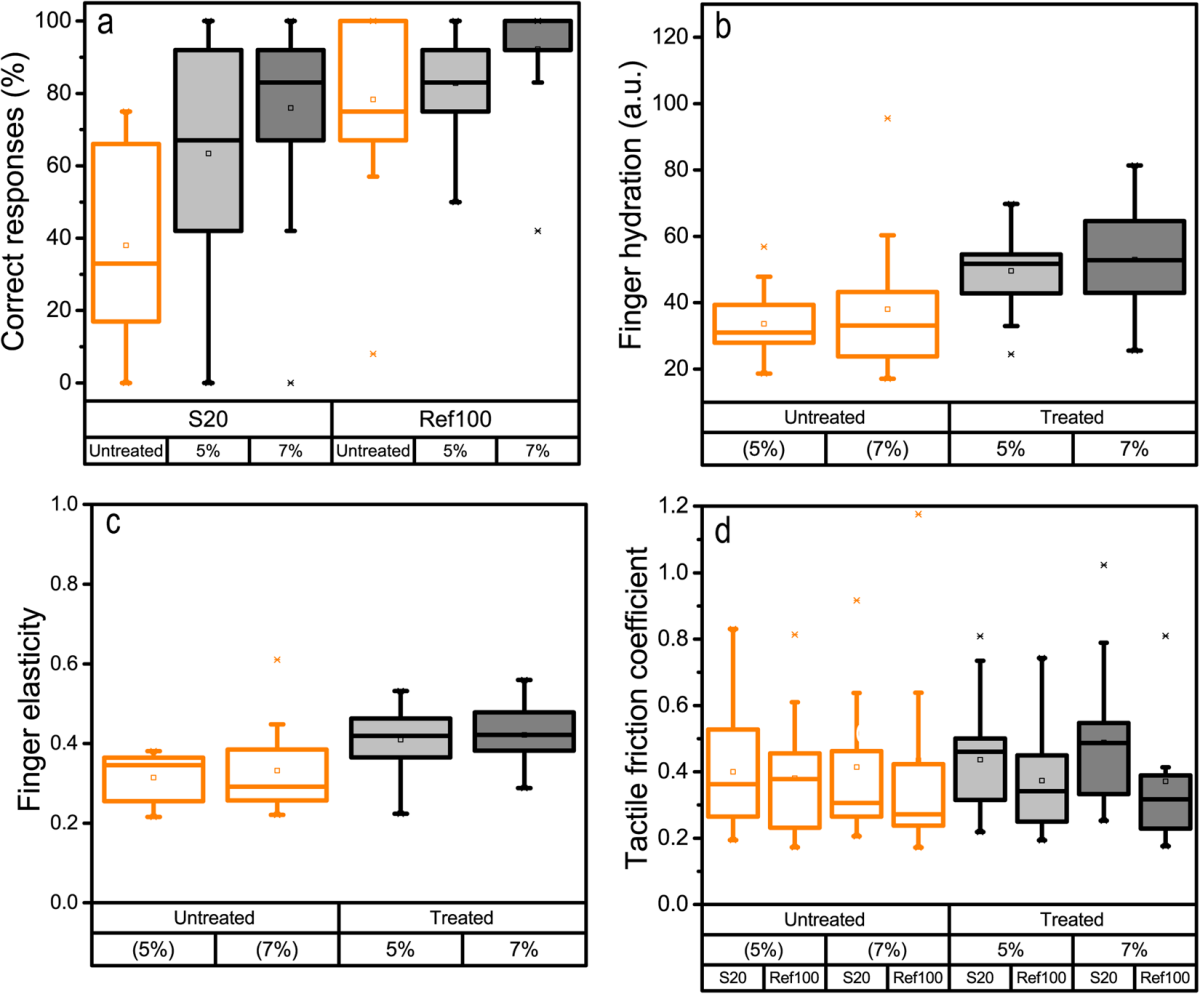
Improvement in tactile discrimination ability with increased finger hydration. Data is shown only for the low performing elderly group (**a**) Improvement in correct responses measured 30 min after application of humectants containing 5% and 7% glycerol (applied on two different days). (**b**) As expected, both humectants increase the finger hydration level. (**c**) Finger elasticity is analogously increased. (**d**) Greater differences in tactile friction between S20 and Ref100 in the treated finger state that could explain the increase in tactile discrimination ability based on greater differences in sticky/slippery feel. Note that when humectant was applied, the physical parameters were measured both before and after. Thus there are “untreated” data which are nonetheless labelled as (5%) and (7%) according to the respective, subsequent treatment.

Two different humectants containing 5% and 7% of the common moisturizer glycerol were applied to the same index finger used in the perception test, on two different days. In the treated state, S20 and Ref100 were repeatedly evaluated. As a check, the high performing group (N = 13) were still high performing after application of 5% glycerol. As can be seen in Fig. 5a, the ability to discriminate S20 significantly improved for the low performing group (F = 15.911, *p* = 0.001 for 5% and F = 18:346, *p* < 0.001 for 7%) after application of humectant to the finger. The interpretation is further strengthened by the fact that the ability to correctly identify Ref100 increased as well, indicating a higher degree of confidence in the assessment. This known humectant also improves the finger hydration of the elderly in the low performing group as can be seen in Fig. 5b. There is a significant increase in both finger hydration and finger elasticity that could explain the increased ability to perceptually distinguish S20 and Ref100. Previous work has indicated that two perceptual mechanisms seem to govern perception, where sticky/slippery has been shown to be the main dimension and can be related to the friction coefficient^10,19^. It has further been established that finger hydration is a major contributor to the individual variations in the absolute values of the friction coefficient^19^. These results thus clearly indicate that hydration and elasticity play a role in the restoration of dynamic tactile discrimination ability of fine texture analogously to static touch on the arm^3^, lips^49^, and index finger^50^. The sticky/slippery perception has been addressed by the instrumental measurements of tactile friction. A significant difference between the tactile friction coefficients measured in untreated and treated states is observed. In the untreated state, no difference at the group level between S20 and Ref100 was noted. However, in the treated state a significant difference between the two surfaces was obtained (F(1,16) = 4.261, p = 0.008 for 5%, and F(1,16) = 4.866, p = 0.003 for 7%), where S20 displays the higher tactile friction. These observations indicate that the improvement in active tactile ability in the low performing group could be related to an improvement in both finger hydration and finger elasticity. This in turn allows the subjects to distinguish the surfaces based on a sticky/slippery perception^10,19^. The improvement effect appears to be both immediate and reversible since a new “untreated” perception test performed one day post application shows that both cutaneous parameter levels and tactile discrimination ability return to the same level as before humectant application.

Hydration is known to modify the properties of the stratum corneum^51–53^ and in part is responsible for a softening thereof. Such deformations in the stratum corneum facilitate pressure stress transmission to the subsurface somatosensory system (touch corpuscles). Friction is also known to increase with the moisture level mainly due to the contribution of skin deformation, in particular at higher pressures. Note that there is no information as to how the topography of the finger print ridges varies with age and between the two elderly groups, but there are no publications indicating bimodal distributions of topography amongst the elderly. Even if topography *were* to vary between any of the groups, it would manifest via the finger friction parameter and would thus effectively be degenerate with the tribological properties.

The simultaneous improvement in the tactile perception discrimination ability and cutaneous parameters upon treatment with glycerol together with the observed differences between young and elderly groups in the dry state indicate that the skin state is important for active tactile perception ability. A comparison of the high and low performing elderly sub-groups in the untreated state however, shows no significant differences in biomechanical or biotribological properties, raising interesting questions as to the mechanisms of the decline in tactile acuity, or rather its *retention by the high performers*. One possibility is that the high performing elderly sub-group has developed a greater (compensating) sensitivity towards another perceptual prompt – for example the vibrations detected in the Pacinian corpuscles^37,38^. The vibration occurs as the finger traverses the periodic structures and its value depends on the wavelength. The sensitivity to these vibrations decreases as the vibration frequency diverges from the optimal sensing frequency of the Pacinian corpuscles^37,38^. The fact that there is no difference in the biomechanical properties of the skin indicates that vibration *transmission* is unlikely to be the difference. The question however remains as to why *two* populations exist and this implicates an additional, neural, contribution to age-related loss of tactile perception. This could, for example, be related to a decrease in density of peripheral nerves and mechanoreceptors in the fingertip, or their ability to transmit signals. Based on the data above it is difficult to draw more conclusions. Thus, a follow up experiment was performed. A new, larger population of elderly participants were screened using the same perceptual discrimination experiment. Two elderly groups were selected (30 each) based on identification as poor performing (in this case less than 60% success) or high performing (>80% success). The density of Meissner Corpuscles (MC) was measured using a microscope as a simple, non-invasive measurement of the somatosensory condition. (Meissner Corpuscles are generally held to be the most important of the mechanoreceptors used in touch and pressure on glabrous skin^54^). It was found that the higher performing group had a statistically higher MC density (see Fig. S8). The average was 2.67 mm^−2^ with a standard deviation of 0.89 mm^−2^. For the lower performing elderly group the average was 1.35 mm^−2^ with SD 0.70 mm^−2^. Thus the lower performance of this group can be confidently linked to a neural decline, though we stress that we do not link the reduced acuity directly to the lower MC density, since there is no data on the other tactile receptors.

### Concluding remarks

There is a clear, statistically significant reduction in the fine texture discrimination with active touch amongst the elderly. Identifying such decline quantitatively is not trivial, but the test presented here, provides a straightforward approach. Not only can the deterioration be measured, but its remediation by training or treatment can also easily be assessed. The reduction in acuity amongst the elderly is matched by a reduction in the biomechanical properties of the skin – moisture, elasticity and finger friction coefficient are all significantly reduced. The finger friction is now known as an important perceptual identifier for fine texture discrimination so this provides a clear pathway for amelioration of the reduced tactile perception.

The observation of that a large proportion of the elderly retain their active touch fine texture discrimination acuity has several important ramifications. At the simplest level, it means that future studies aimed at remediating tactile discrimination need to take this into account. More importantly, this observation casts significant light on the mechanism of the decline in acuity. Since the biomechanical decline of the skin properties is indistinguishable for these two populations, the reduced performance cannot be associated directly with the age related changes in elasticity, moisture and friction. This almost certainly rules out the sticky-slippery perceptual dimension as the prompt for the performance. Another dimension is associated with vibrations detected by Pacinian and/or Meissner Corpuscles. Since the mechanical skin properties in the two groups are comparable, the vibration transmission itself can be cautiously ruled out, which strongly implies that the difference between the two groups has a neural decline associated with receptor sensitivity, density, or signal transmission. A reduced density of receptors has clearly been shown for the poor performers. Thus, age-related decline in neural properties is the primary explanation for the reduction in active touch acuity. Nonetheless, an improvement in the biomechanical properties of the skin through rehydration, for example as performed here, means that the loss in discrimination in the one dimension, can be significantly ameliorated by an improvement in tactile discrimination ability in the other, friction based, dimension.

## Methods

### Wrinkle-patterned model surfaces

Six wrinkled surfaces were used in the initial perceptual test and were manufactured in-house based on a surface wrinkling technique presented earlier^10,55^. They consist of UV-curable adhesive polymer NOA81 (Norland Optical Adhesive 81, Norland Products Inc., Cranbury, USA) and were templated from sinusoidal textures imposed on a PDMS surface (polydimethoxysiloxane, Sylgard 184 Dow Corning, USA), with nominal wavelengths according to previous recipes^19^ ~20 µm (S20), ~40 µm (S40), ~60 µm (S60), ~80 µm (S80) and ~100 µm (Ref100). A blank reference surface was replicated against smooth, unwrinkled PDMS. See Figs S3 and S4 profilometry results.

### Tactile discrimination test

A same-different paradigm was used, *i.e*. the task was to judge whether two surfaces were perceived identical or not. The method of constant stimuli was employed for the presentation of stimuli, *i.e*. the participants were always presented with Ref100 first, followed by the test stimuli and were instructed to judge whether the second surface was perceived as the same as or different from the reference. Each surface was compared with the reference surface six times in a randomized order, resulting in 36 comparisons in total for each participant in the untreated state. The instruction was to stroke the surface back and forth with the index finger of the dominant hand. The perception test was performed blind-folded. All subjects were allowed to practice the test procedure on the same pairs of surfaces prior to the test. The surfaces were cleaned with acetone both during and after each test series.

### Subjects and experimental conditions

30 elderly women (mean: 73 ± 4.5 years; range 67–85) and 30 young women (mean: 22 ± 1.5 years; range 19–25) participated in the study. Each participant had a unique randomized order of surface presentation for perception tests and tactile friction measurements. The study was performed in ambient indoor air, where the mean temperature was 23 ± 1.3°C and the relative humidity 53 ± 8.7%.

### Inclusion criteria, ethics and guidelines

The inclusion criteria were addressed during the recruitment process, and were also checked at the start of the experimental session. None of the participants had any skin disease, neurological disease, diabetes or were allergic to cosmetic products. None of the participants were pregnant or had been breastfeeding the last six months. Signed, informed consent was given and the subjects were informed that they could quit the experiment at any time if they so wished. All procedures were performed in accordance with the ethical standards of the 1964 Helsinki declaration and its later amendments of ethical standards regarding studies with human participants. The protocols are as for reference^10^, and were approved by the Research Committee of the Institute for Surface Chemistry and the Ethics Committee of Stockholm University.

### Cutaneous measurements

Cutaneous status or biomechanical properties were measured by means of different probes from Courage & Khazaka Electronic GmbH (Cologne, Germany). Finger hydration was measured with a Corneometer CM 825 in arbitrary units (a.u.), based on electrical capacitance. Each individual moisture value is an average (mean ± standard deviation) of five repeated measurements on the index fingertip of the dominant hand. Finger elasticity was measured on the index finger of the dominant hand with a Cutometer MPA 580 by measuring the vertical deformation of the skin when pulled into a 2 mm diameter probe with an optical sensor. Each measurement consisted of three suction cycles of 2 s using a constant negative pressure of 450 mbar, followed by a 2 s period when the pressure was switched off (relaxation phase) allowing the skin to return to its original shape. The elastic recovery parameter (also called net elasticity), R5 (Ur/Ue) is used to represent finger elasticity in this work obtained, as it has been identified as a suitable parameter for comparing difference between young and aged skin^41^. The different parameters from the time/strain curves (elasticity curves) are described in supplementary information, the parameters Ue and Ur refer to the linear elastic response on deformation and relaxation respectively.

### Tactile friction measurements

Finger friction (or tactile friction) was measured with a ForceBoard (Industrial Dynamics AB, Sweden), a universal friction and force tester equipped with one horizontal and one tangential load cell, individually connected to the same plate of assembly. Upon interaction, a mechanical load is converted into voltage signals that are amplified and proportional to the applied load in N. The tangential force (friction force), and vertical force (applied load) were continuously recorded using DAQFactory software at a rate of 100 Hz as a finger was moved over the surfaces. Friction coefficients were calculated at each data point as the ratio of the friction force and applied load the average dynamic friction coefficients of ten stroking cycles were calculated and compared^19^. (Note that here “friction coefficient” refers to the macroscopic interaction of the entire finger-textured surface contact, rather than the local, “asperity contact” shear stress which should be invariant since the surfaces are all the same material). The surfaces S20, S60 and Ref100 were measure in triplicate in each experimental session (randomized order). The participants were instructed to use their preferred load, speed and angle of the index finger the same for all the measurements. The surfaces were measured in randomized order.

### Tests after application of a humectant

To assess the effects of skin remediation on perception, the 30 elderly participants performed a second perception test after application of a humectant. Two different humectant systems, were used in which the active moisturizing ingredient in both cases was glycerol at concentrations of 5% and 7%, respectively. Two humectant systems, with different carrier compositions were employed to limit potential artefacts from the non-humectant components. The two different systems were tested on two different days, and their order randomized. The humectant was applied with three fingers (including the index finger) onto the cheek (as a counter surface to distribute the material evenly and in a manner perceived as “natural” by the participants). The cutaneous parameters and tactile friction were also measured in the treated state on the index finger.

### Meissner Corpuscle density

Meissner corpuscles (MC) densities were obtained according to an established protocol described and referenced in the ESI. Optical images were obtained for each subject by sampling a 2,5 × 2 mm area over the midpoint of the volar aspect of the distal phalanx of Digit I, on the dominant hand. An *in vivo* reflectance Confocal Microscope (RCM) (Vivascope 1500, Lucid Inc., NY) was used to obtain all the images at a specific depth. A fuller description is provided in the Supplementary Information.

### Statistical analysis

One-way ANOVAs or two-way repeated ANOVAS have been done with Origin (physical data) and SPSS (perception data) to test significance between variables, *i.e*. the effect of age, effect of humectant and effect of different concentrations of glycerol. The Alpha level for statistically significance was set to *p* < 0.05. Due to a wide spread in absolute values obtained when measuring tactile perception ability with human subjects as well as properties on the skin *in vivo*, we show all results as box plots, showing the interquartile range IQR (25th and 75th quartile = 50% of the data) with the mean (square), median (line), whiskers indicating variability within 1.5 IQR, as well as outliers. The age differences in the ability to correctly identify S20 and the ability to correctly detect S40 were analysed in separate one-way ANOVAs. Tukey *post hoc* analyses were used to identify which of the conditions were significantly different.

## Electronic supplementary material

Supplementary Information
