# Supplementary material for: Mechanisms of tactile sensory deterioration amongst the elderly
Source: Sci Rep. 2018 Apr 19;8:5303. doi: 10.1038/s41598-018-23688-6 (PMC5908919; doi:10.1038/s41598-018-23688-6)
Supplement: Supplementary file 1 — Supplementary Information [file 41598_2018_23688_MOESM1_ESM.pdf]

Supplementary Information

Mechanisms of tactile sensory deterioration amongst the elderly.

Lisa Skedung[1], Charles El Rawadi[2], Martin Arvidsson[1], Céline Farcet[2], Gustavo S. Luengo[2,*] Lionel Breton[2], and Mark W. Rutland[1,3,*]

[1]RISE Research Institutes of Sweden, Bioscience and Materials, Stockholm, SE-114 28, Sweden

[2]L'Oréal Research and Innovation, Aulnay-sous-Bois, 93600, France

[3]KTH Royal Institute of Technology, Surface and Corrosion Science, Stockholm, SE-100 44, Sweden

*Corresponding author: mark@kth.se

*Corresponding author: gluengo@rd.loreal.com

This PDF file includes Supplementary Information and Supplementary Figures S1-S10.

It describes the skin elasticity, the wrinkled surface characterisation and more details of the test procedure and somatosensory cell density analysis. Additional biomechanical results are shown. A comparison of the load dependence is also included, since this impacts mechanisms of perception in general.

1. Supplementary Information

1.1. Skin elasticity

The Cutometer MPA 580 (Courage & Khazaka Electronic GmbH, Köln, Germany) was used to measure skin elasticity. It measures the vertical deformation of the skin, in mm, when pulled into the opening of a probe (2 mm in diameter) and the response of the skin when it is released[1]. Each measurement consisted of three suction cycles of 2 s using a constant negative pressure of 450 mbar, followed by a 2 s period when the pressure was switched off (relaxation phase) allowing the skin to return to its original shape[2,3]. A typical skin deformation curve is shown in Fig. S1. Many parameters describing the elastic and viscoelastic properties of the skin were obtained from the time/strain curves (elasticity curves), and the definitions of evaluated parameters are shown in Table S1[1,3].

A comparison of dry state finger elasticity of young and elderly groups (Elderly N=26, mean: 73±4.5 years; range 67-85 and for the young group N=29, mean: 22±1.5 years; range 19-25) was performed. The measured elasticity parameters for the young (Y) and elderly (E) groups are compared in BOX plots in Fig. S2. All evaluated parameters are shown in the same plot, although some are relative measures (R2, R5, R6 and R7) and some are measured in mm (R0, R1 and R8)[1,3,4]. The significant differences of the means between young and aged skin for each parameter were analysed with one-way ANOVAS, and the p-values are listed in Table S2. As can be seen, there are significant differences in many of the elasticity parameters between young and aged skin in the index. The results show that aged skin is less elastic compared to young skin when looking at parameters R2, R5 and R7 (a value of 1 means that the skin is 100% elastic). A higher R0- and R1-value indicates more initial skin deformation and less firm skin which appears to be the case for more aged skin.

1.2. Characterization of wrinkled surfaces

The surface wavelength was visualized and quantified with a stylus profilometer (DektakXT Profiler, Nano GmbH, Germany) on the templated wrinkled surfaces [5]. Line scans of 1.1 mm as well as area images of 1x1 cm acquired from 250 lines obtained with a stylus (radius 2 µm) were obtained by moving the stylus tip across the wrinkles. The wavelength was obtained from the stylus analysis and the parameter *PSm* - the average peak spacing. The force of the stylus on the surface was set to 3 mg. Data analyses were made in the Vision64 software program. An example of a 3D visualization is shown in Fig. S3 for surface Ref100. The wavelengths extracted are shown in Fig. S4. The same wavelengths are measured (within error) on surfaces that have been used in testing, confirming that the wrinkles are robust towards repeated touching.

1.3. Comparing the skin status of low and high performing elderly

Since there was an increased hydration, elasticity and tactile friction as well as tactile perception with the use of humectant, it was of interest to compare the bio-tribological and bio-mechanical properties between the low and high performing elderly participants to determine whether this could explain the differences in tactile perception performance between the two groups. The comparison of finger hydration (**Fig. S3**) indicates no statistical difference between low and high performing elderly subjects (p=0.915). The parameter R5 is compared between the high and low performers in Fig. S4 (p=0.227). As with the other elasticity parameters, *i.e.* R0, R1, R2, R6, R7 and R8, no significant difference in the average elasticity was observed between high and low performers. The measured tactile friction in the untreated/dry state has also been evaluated for the two groups of low and high performers in Fig. S5. Three surfaces were used, *S20, S60 and Ref100.* The high performing group show greater individual differences with higher maximum levels of tactile friction, as compared to the low performing group.

However, no statistically significant difference between the two groups or between *the three surfaces* was obtained.

1.4. Meissner Corpuscles quantification

Meissner corpuscles (MC) images were obtained according to established protocols[6-9] by sampling a 2.5 x 2 mm area over the midpoint of the volar aspect of the distal phalanx of Digit I, on the dominant hand. An *in vivo* reflectance Confocal Microscope (RCM) (Vivascope 1500, Lucid Inc., NY) was used to obtain all the images at a specific depth. The procedure started by attaching a stainless-steel ring with a specific disposable polycarbonate window to the skin by using a drop of Crodamol STS (Croda, USA) to guarantee the contact. The ring was then connected to the microscope lens by using normal ultrasound contact gel (Aquasonic 100, Parker Laboratories, USA). A stack of images from the surface to the dermal interface were obtained to visualize the depth at which the dermal papillae appear in each subject. After the calculation of the optimal depth, a mosaic of 6 x 5 images (corresponding to 2,5x2 mm) adjacent images were obtained at that specific horizontal plane. Two sequential mosaics were performed in order to eliminate interferences from red blood cells passing in the capillary loop that also appear in the papillae.

The 6x5 images mosaics were examined first automatically, by a specific image analysis routine to increase the contrast of the images and to remove the interference of the blood in the dermal papillae, to observe the Meissner corpuscles more easily (code written in Java and implemented in ImageJ, NIH, USA). After the first automatic evaluation a technician confirmed all the images in order to validate the number of corpuscles in the mosaic. The main criterion to identify Meissner corpuscles was the dimension (30-90 μm), existence of only one MC in the papillae and absence of movement in the two sequential mosaic images. Results were expressed in density of MC (nº/area of evaluation).
The consistency of the study analysis was obtained by guaranteeing that the technician who performed the images was always the same. Also, the technician who performed the image analysis was also the same, but different from the first one. The image analysis was performed in blind in order to avoid any bias of the results.

2. Supplementary Figures and Tables

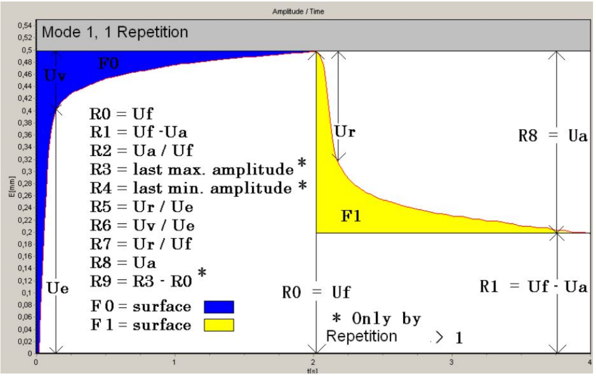

Figure S1. A typical skin deformation versus time plot obtained with the Cutometer. One measurement consisted of three repeated suction/relaxation curves. This figure is used by permission from Courage - Khazaka Electronic.

Table S1. Evaluated parameters from the skin deformation versus time plot.

Parameters		
Skin distensibility	Uf	R0
Residual deformation	Uf–Ua	R1
Gross elasticity	Ua/Uf	R2
Net elasticity	Ur/Ue	R5
Ratio of delayed-to-immediate distension	Uv/Ue	R6
Ratio of elastic recovery to distensibility	Ur/Uf	R7
Total recovery	Ua	R8

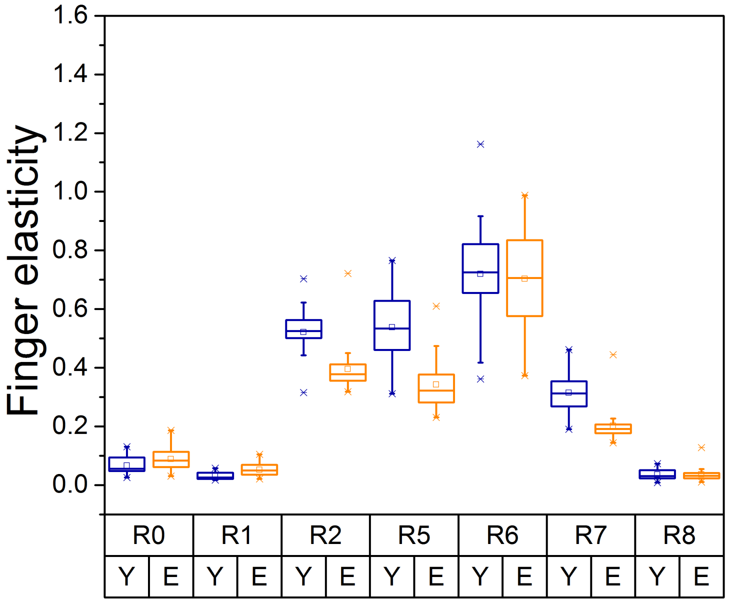

Figure S2. BOX plot of elasticity parameters measured in the index finger. Comparison of different finger elasticity parameters between the young (N=29) and elderly groups (N=26). R0, R1 and R8 are measured in mm whereas R2, R5, R6 and R7 are relative parameters.

Table S2. Summary of elasticity results. The significant differences when comparing the means of young and aged skins are summarized by the corresponding p-values obtained from one-way ANOVAS.

	**R0**	**R1**	**R2**	**R5**	**R6**	**R7**	**R8**
**Finger**	**0.024**	**<0.001**	**<0.001**	**<0.001**	0.713	**<0.001**	0.855

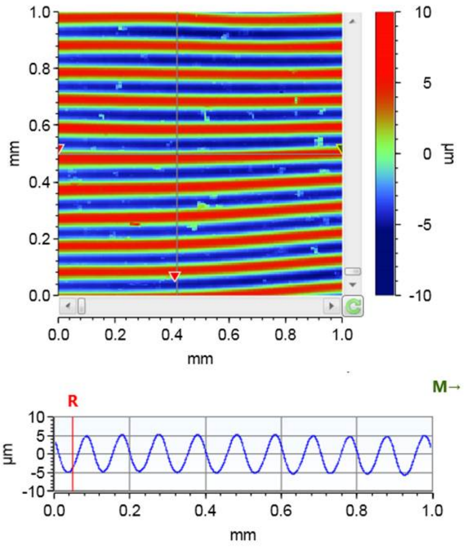

Figure S3. An example of a 3D visualization obtained with a stylus profilometer on Ref100. The quantified wavelength is obtained from the PSm parameter, *i.e.* the average peak spacing.

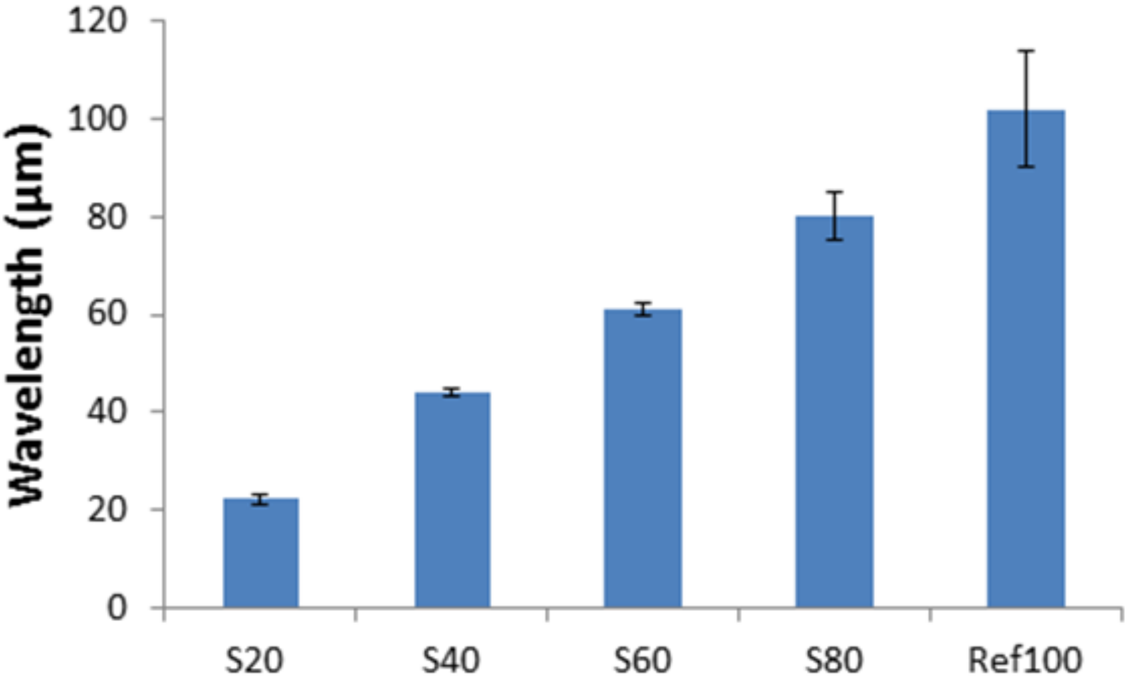

Figure S4. Wrinkle wavelength obtained from line scans with a stylus profilometer and the average spacing parameter PSm (N=3)

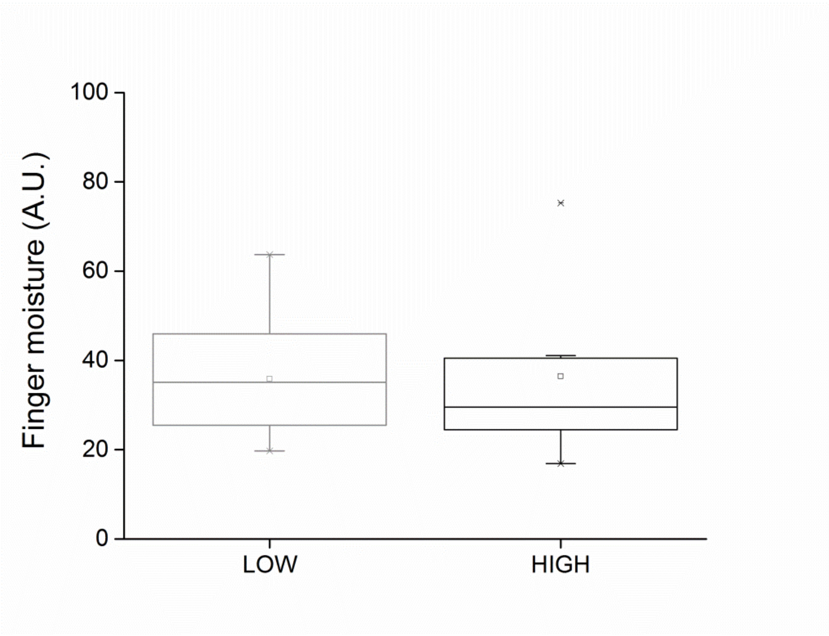

Figure S5. Comparison of finger moisture of the low and high performing groups.

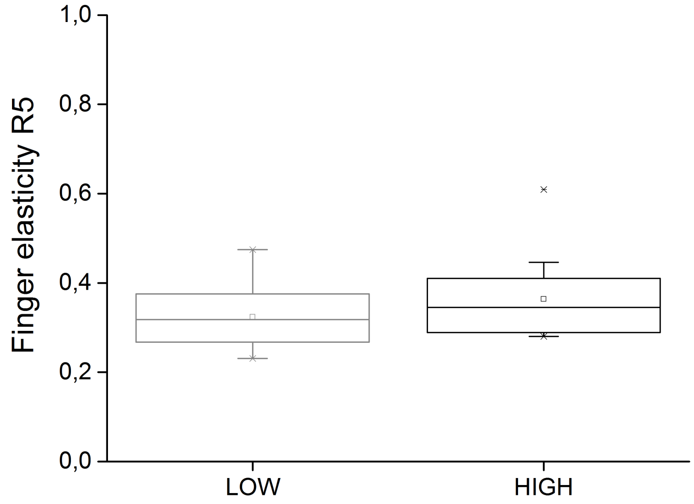

Figure S6. Comparison of finger elasticity (parameter R5) of the elderly low and high performers. No difference in any of the other evaluated elasticity parameters were observed.

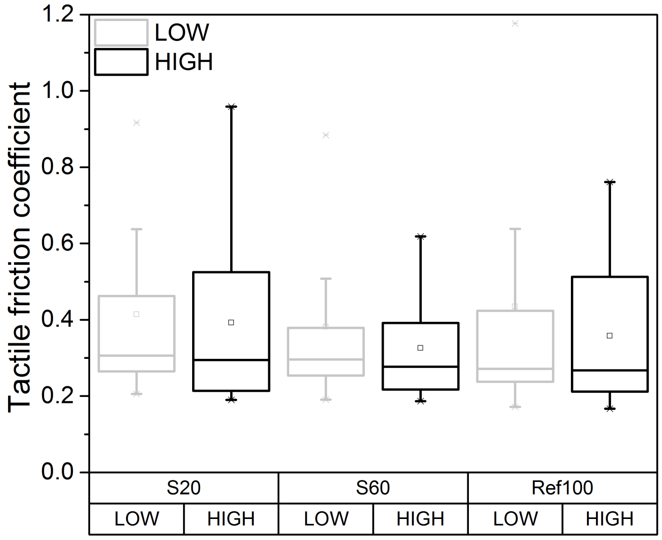

Figure S7. Comparison of tactile friction coefficients of the elderly low and high performers measured on surfaces S20, S60 and Ref100.

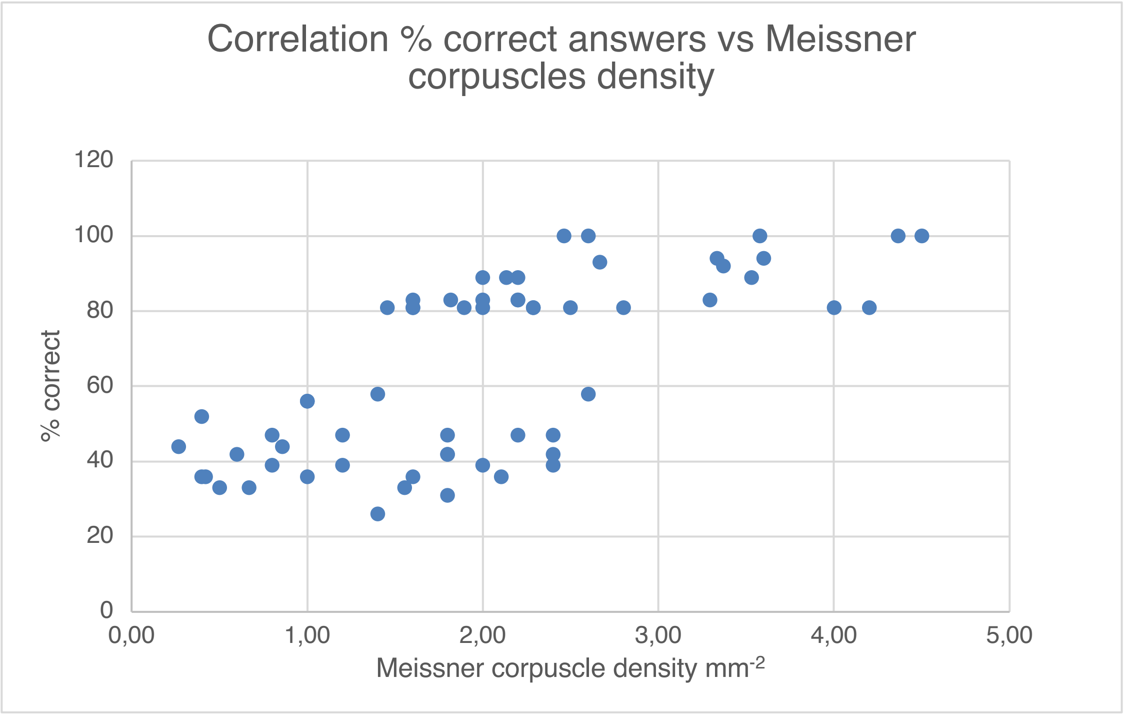

Figure S8. Percentage correct answers as a function of MC density for two larger groups (each N=30) who were analysed at a later stage to characterize the two types of behaviour observed in the elderly group. While there is overlap, it is clear that the lower performing group have a considerably lower average density of MC. Note that in this subsequent study, low performers were defined as having less than 60% success in the test, whereas high performers scored better than 80%.

Applied Load- Relation to friction coefficient and perceptual sensitivity.

In the introduction of the paper we have discussed the fact that while the friction coefficient between the finger and a surface is the physical parameter which distinguishes different surfaces, the load is unconsciously regulated to maintain an optimal friction force. In which case it is worth addressing whether the various groups modify the load (how hard they press) in response to the different friction coefficients. If there were different trends between the different groups then this could have perceptual implications. Fig S9 and Fig S10 show the differences in load between the young and elderly groups and the high and low performing groups respectively. In neither case are there statistically significant differences between the groups, though it can be seen that the young do press less hard (higher friction) as predicted and also have a lower standard deviation. The high performing elderly group also display a lower standard deviation which may also be significant to a future understanding of perceptual mechanisms.

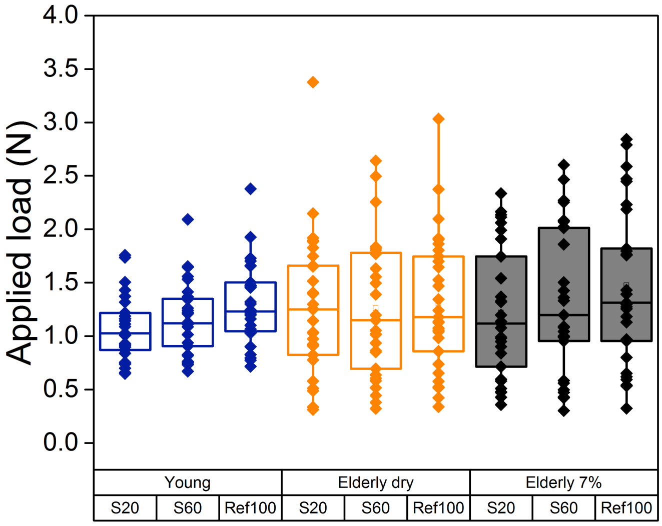

Figure S9. Average loads employed by the young and elderly groups employed during the friction coefficient measurements on the 3 surfaces S20, S60 and Ref100. In general the standard deviation is smaller for the higher friction (lower applied load) surfaces.

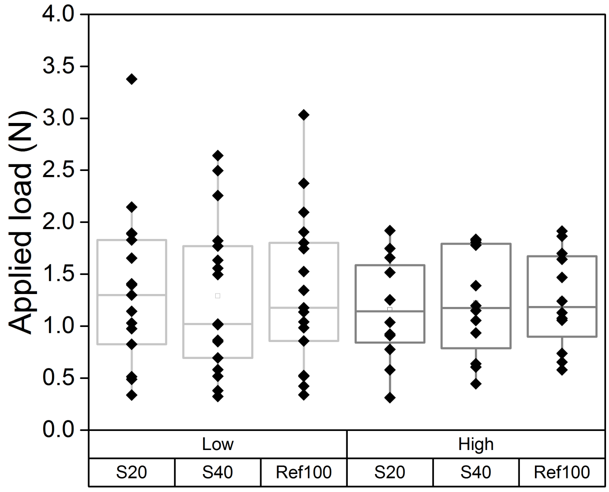

Figure S10. As for S9 but comparing the high and low performing elderly groups. The high performing groups display a lower standard deviation.

3. Supplementary References

1 Dobrev, H. In vivo noninvasive study of the mechanical properties of the human skin after single application of topical corticosteroids. *Folia Med (Plovdiv)* **38**, 11-17 (1996).

2 Kim, E., Cho, G., Won, N. G. & Cho, J. Age-related changes in skin bio-mechanical properties: the neck skin compared with the cheek and forearm skin in Korean females. *Skin Res Technol* **19**, 236-241, doi:10.1111/srt.12020 (2013).

3 Ryu, H. S., Joo, Y. H., Kim, S. O., Park, K. C. & Youn, S. W. Influence of age and regional differences on skin elasticity as measured by the Cutometer. *Skin Res Technol* **14**, 354-358, doi:10.1111/j.1600-0846.2008.00302.x (2008).

4 Krueger, N., Luebberding, S., Oltmer, M., Streker, M. & Kerscher, M. Age-related changes in skin mechanical properties: a quantitative evaluation of 120 female subjects. *Skin Res Technol* **17**, 141-148, doi:10.1111/j.1600-0846.2010.00486.x (2011).

5 Chung, J. Y., Youngblood, J. P. & Stafford, C. M. Anisotropic wetting on tunable micro-wrinkled surfaces. *Soft Matter* **3**, 1163-1169, doi:10.1039/b705112c (2007).

6 Herrmann, D. N., Boger, J. N., Jansen, C. & Alessi-Fox, C. In vivo confocal microscopy of Meissner corpuscles as a measure of sensory neuropathy. *Neurology* **69**, 2121-2127, doi:10.1212/01.wnl.0000282762.34274.94 (2007).

7 Almodovar, J. L. *et al.* In vivo confocal microscopy of Meissner corpuscles as a novel sensory measure in CMT1A. *Journal of the Peripheral Nervous System* **16**, 169-174, doi:10.1111/j.1529-8027.2011.00342.x (2011).

8 Dillon, Y. K., Haynes, J. & Henneberg, M. The relationship of the number of Meissner's corpuscles to dermatoglyphic characters and finger size. *J Anat* **199**, 577-584 (2001).

9 Ridley, A. Silver staining of nerve endings in human digital glabrous skin. *Journal of Anatomy* **104**, 41-48 (1969).
